# Structural conservation among variants of the SARS-CoV-2 spike postfusion bundle

**DOI:** 10.1073/pnas.2119467119

**Published:** 2022-04-01

**Authors:** Kailu Yang, Chuchu Wang, K. Ian White, Richard A. Pfuetzner, Luis Esquivies, Axel T. Brunger

**Affiliations:** ^a^Department of Molecular and Cellular Physiology, Stanford University, Stanford, CA 94305;; ^b^Department of Neurology and Neurological Sciences, Stanford University, Stanford, CA 94305;; ^c^Department of Structural Biology, Stanford University, Stanford, CA 94305;; ^d^Department of Photon Science, Stanford University, Stanford, CA 94305;; ^e^HHMI, Stanford University, Stanford, CA 94305

**Keywords:** cryogenic electron microscopy, membrane fusion, SARS-CoV-2, COVID-19, HR1HR2

## Abstract

Variants of severe acute respiratory syndrome coronavirus 2 (SARS-CoV-2) challenge currently available COVID-19 vaccines and monoclonal antibody therapies due to structural and dynamic changes of the viral spike glycoprotein (S). The heptad repeat 1 (HR1) and heptad repeat 2 (HR2) domains of S drive virus–host membrane fusion by assembly into a six-helix bundle, resulting in delivery of viral RNA into the host cell. We surveyed mutations of currently reported SARS-CoV-2 variants and selected eight mutations, including Q954H, N969K, and L981F from the Omicron variant, in the postfusion HR1HR2 bundle for functional and structural studies. We designed a molecular scaffold to determine cryogenic electron microscopy (cryo-EM) structures of HR1HR2 at 2.2–3.8 Å resolution by linking the trimeric N termini of four HR1 fragments to four trimeric C termini of the Dps4 dodecamer from *Nostoc punctiforme*. This molecular scaffold enables efficient sample preparation and structure determination of the HR1HR2 bundle and its mutants by single-particle cryo-EM. Our structure of the wild-type HR1HR2 bundle resolves uncertainties in previously determined structures. The mutant structures reveal side-chain positions of the mutations and their primarily local effects on the interactions between HR1 and HR2. These mutations do not alter the global architecture of the postfusion HR1HR2 bundle, suggesting that the interfaces between HR1 and HR2 are good targets for developing antiviral inhibitors that should be efficacious against all known variants of SARS-CoV-2 to date. We also note that this work paves the way for similar studies in more distantly related viruses.

## Significance

Emergence of viral pathogens necessitates new approaches to study viral fusion and entry into host cells. A key step in mediating fusion involves the formation of a six-helix bundle within the spike protein. Rapid structural characterization of this state has been difficult, hindering understanding of emerging variants. We developed a method to efficiently determine high-resolution bundle structures by molecular scaffolding and cryogenic electron microscopy. Using this method, we determined bundle structures of severe acute respiratory syndrome coronavirus 2 (SARS-CoV-2) variants. These structures reveal local effects of mutations on HR1HR2 interactions but global conservation of the bundle architecture among SARS-CoV-2 variants. We predict that inhibitors disrupting the postfusion bundle might be broadly efficacious against variants and even more distantly related lethal viruses.

Three previously unknown beta-coronaviruses have emerged in the first two decades of this century: severe acute respiratory syndrome coronavirus (SARS-CoV) in 2003, Middle East respiratory syndrome coronavirus (MERS-CoV) in 2012, and severe acute respiratory syndrome coronavirus 2 (SARS-CoV-2) in late 2019 (1). The most recent outbreak of SARS-CoV-2 that causes coronavirus disease 2019 (COVID-19) has claimed about 6 million lives in 2 y, and several variants of concern have emerged around the globe despite the relatively low mutation rate of coronaviruses (2). Some of these variants pose a challenge to currently available vaccines (3–6), likely due to structural changes of the target of these vaccines (7–11). Hence, there is an urgent need for new antiviral therapeutics (12) that target regions of viruses with conserved structural features that are less likely to be affected by mutations.

**Fig. 1.**
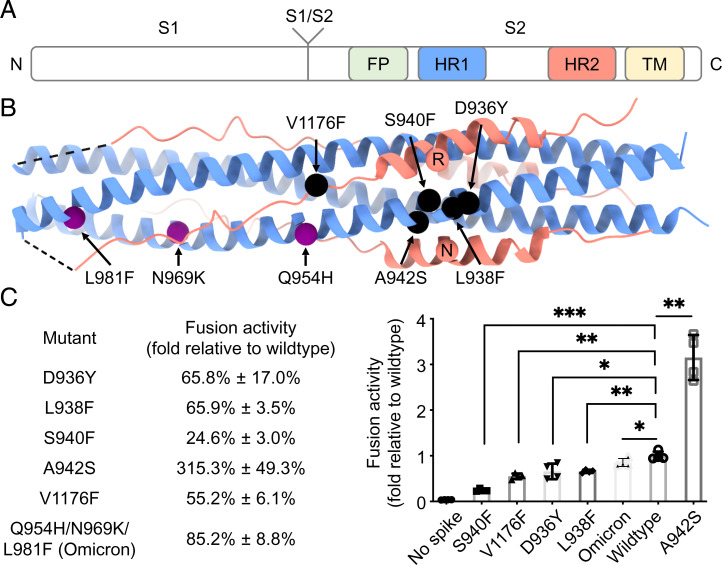
Mutations of interest in the HR1HR2 bundle of SARS-CoV-2 variants. (*A*) Schematic diagram of the domain structures of the SARS-CoV-2 spike protein. The N and C termini are labeled on the left and right, respectively. FP, fusion peptide; HR1, heptad repeat 1; HR2, heptad repeat 2; TM, transmembrane region. (*B*) Locations of the five selected point mutations of SARS-CoV-2 variants (black spheres) and the three mutations of the SARS-CoV-2 Omicron variant (purple spheres) indicated in the crystal structure of the HR1HR2 bundle (PDB ID code 6lxt). Two HR2 residues, R1185 and N1187, that may be affected by the selected mutations are shown as red spheres. The HR1 and HR2 fragments are colored as light blue and light red, respectively. (*C*) Effects on fusion activity of these mutations. The fusion activity is shown as a percentage (*Left*)/fold change (*Right*) relative to that of the wild type (*Materials and Methods*). The Omicron construct used here for the fusion assay has three mutations—Q954H, N969K, and L981F—in the HR1 portion of the HR1HR2 bundle, but not other mutations from different regions of the spike found in the Omicron variant. *P < 0.05, **P < 0.01, ***P < 0.001, by a Student’s *t* test.

SARS-CoV, MERS-CoV, and SARS-CoV-2 are enveloped viruses that rely on membrane fusion to deliver RNA to the host cell (13). In each case, the process of viral membrane fusion (14, 15) is mediated by the trimeric viral spike glycoprotein (S) that is cleaved into S1 and S2 subunits by multiple host proteases upon infection (16) (Fig. 1*A*). S1 recognizes the human angiotensin-converting enzyme 2 (ACE2) receptor and dissociates from S2. Subsequently, S2 undergoes substantial conformational changes that drive membrane remodeling. Similar to other enveloped viruses (14, 15), this process likely proceeds via an intermediate extended state that pulls together the two membranes via the transmembrane domain and fusion peptide of the S2 subunit (17). Two heptad repeat regions, HR1 and HR2, distant from each other in the prefusion S, drive membrane fusion by assembly into a six-helix bundle (18). This HR1HR2 bundle formation is thought to provide the energy for membrane fusion and is therefore a target for therapeutics, as exemplified by peptide inhibitors that disrupt infection by the HIV-1 (19, 20), SARS-CoV (21), MERS-CoV (22), SARS-CoV-2 (23–25), human parainfluenza virus 3 (26), and respiratory syncytial virus (26).

Despite the established value of inhibitors targeting formation of the HR1HR2 bundle, the structural plasticity of this bundle upon mutation is largely unknown. Comparison with distantly related viruses suggests that the overall architecture is maintained despite vast differences in primary sequence (*SI Appendix*, Fig. S1). To what degree does the structure of the HR1HR2 bundle change upon mutation? To address this question, we surveyed mutations of all currently known variants (including Omicron) of SARS-CoV-2 S in the postfusion HR1HR2 bundle, selected eight mutations of potential interest, and investigated their effects on structure and function.

Structural characterization of the HR1HR2 bundle has proven surprisingly challenging. To date, two successful approaches for determining structures of the HR1HR2 bundle have been employed. First, several HR1HR2 structures with the HR1 and HR2 domains synthetically linked were determined by X-ray crystallography (2.9 Å, Protein Data Bank [PDB] ID code 6lxt; 1.5 Å, PDB ID code 6m1v) (23, 25). Second, a sample of postfusion S2 was generated from a recombinant source (mammalian HEK-293F cells) expressing full-length S; as such, multiple states of S undergoing spontaneous transition from the prefusion to the postfusion state were present in the sample and the postfusion structure was determined by single-particle cryogenic electron microscopy (cryo-EM) (3.0 Å, PDB ID code 6xra) (18). Although the structures of the postfusion HR1HR2 bundle are similar, there are differences between these structures and the local resolution is quite variable or limited. More importantly, neither approach is particularly suited for efficient structure determination of multiple mutants at high resolution. Therefore, we decided to develop a platform for using single-particle cryo-EM to efficiently determine structures of HR1HR2 bundles at atomic resolution.

The postfusion HR1HR2 bundle of SARS-CoV-2 is a 115 × 25 × 25 Å bundle consisting of six helices (PDB ID code 6lxt) (23). Its molecular weight is 40 kDa, close to the theoretical minimum size needed to achieve a reconstruction with near-atomic resolution by cryo-EM (27). To our knowledge, it has not yet been possible to determine structures of individual proteins <50 kDa to high resolution, with exception in the case of multimers (28, 29) or small RNA molecules (30). In addition, efforts extending the resolution limit of cryo-EM have largely focused on globular proteins (28, 29, 31, 32), perhaps because fibrous samples are more flexible, more susceptible to the issue of preferred orientation, and require thicker ice to bury the entire particle—all of which inevitably increase noise in the already extremely low-contrast and hard-to-align images. To overcome the size limit of single-particle cryo-EM, two strategies have been employed to increase the effective mass of the target protein, e.g. using antibodies/nanobodies/legobodies (33, 34) and molecular scaffolds (35–38). Since developing antibodies/nanobodies/legobodies can be time-consuming we resorted to the molecular scaffold approach. We first attempted to use existing scaffolds but were unable to engineer a linkage ensuring proper HR1HR2 bundle formation. We therefore designed a scaffold to efficiently determine structures of the postfusion HR1HR2 bundle and its mutants to near-atomic resolution by single-particle cryo-EM.

Our high-resolution wild-type structure of the HR1HR2 bundle resolves uncertainties in some side-chain positions present in prior structures. Our HR1HR2 structures of SARS-CoV-2 variants reveal an overall architecture that is highly conserved, with only side-chain rearrangement for five point mutations and, for the Omicron variant containing three mutations in HR1, a slight shift of the HR2 backbone in a nonhelical region that interacts with HR1. These results suggest that interactions between HR1 and HR2 are excellent targets for disruption by broadly efficacious antiviral inhibitors. Moreover, our approach can be directly used to study the binding of potential HR2-based peptide inhibitors and adapted to study the postfusion bundles of other coronaviruses or other structurally similar viruses.

## Results

### Identification of Candidate Mutations in the HR1HR2 Bundle.

We surveyed naturally occurring mutations in the postfusion HR1HR2 bundle from the SARS-CoV-2 spike variants database (39, 40) and selected five mutations of potential interest, initially based on a crystal structure of the HR1HR2 bundle (PDB ID code 6lxt) (23), for further characterization (Fig. 1*B*). We selected HR1 D936Y because D936 forms a salt bridge with the HR2 residue R1185, HR1 S940F because S940 is near the D936/R1185 salt bridge and mutation from a small serine to a larger phenylalanine might cause a steric clash with that salt bridge, HR1 L938F because L938 is located in the center of the helical bundle and mutation from a small leucine to a larger phenylalanine would occupy more space and potentially fill a hole within the bundle, HR2 V1176F because V1176 is hydrophobic but exposed to solvent despite not being involved in HR1HR2 interaction in the wild-type structure, and HR1 A942S because the serine might interact with the HR2 residue N1187 by hydrogen bonding. Although the above structure-based selection was performed before the World Health Organization began to designate variants of concern (VOC), the Gamma VOC indeed contains the selected V1176F mutation (*SI Appendix*, Table S1). In addition, we included a triple mutant corresponding to the Omicron VOC with Q954H, N969K, and L981F in the HR1 part of the HR1HR2 bundle. Note that the Omicron variant in this study refers to the Omicron BA.1 subvariant and that the Omicron BA.2 and BA.3 subvariants only contain the Q954H and N969K mutations but not the L981F mutation. Mutations in the HR1HR2 bundle of other VOCs, including S982A in the Alpha VOC and D950N in the Delta VOC, appear unlikely to induce a structural change of the HR1HR2 bundle. The sequence alignment of the wild-type HR1HR2 as well as the five single-mutants and the Omicron triple-mutant is shown in *SI Appendix*, Fig. S2.

### Effects of Mutations on the Fusion Activity.

We used a validated cell–cell fusion assay (8, 18) to measure the fusion activities of the five HR1HR2 single mutants and the Omicron HR1HR2 triple mutant of S and compared them to that of the wild-type S (Fig. 1*C*). The fusion activities of the D936Y, L938F, and V1176F mutations are ∼60% of that of wild type, while the fusion activity of the S940F mutant is ∼25% of that of wild type. In contrast, the fusion activity of the A924S mutation increases approximately threefold. The fusion activity of the Omicron triple mutant is ∼80% of that of wild type.

### A Molecular Scaffold to Determine Structures of the HR1HR2 Bundle.

**Fig. 2.**
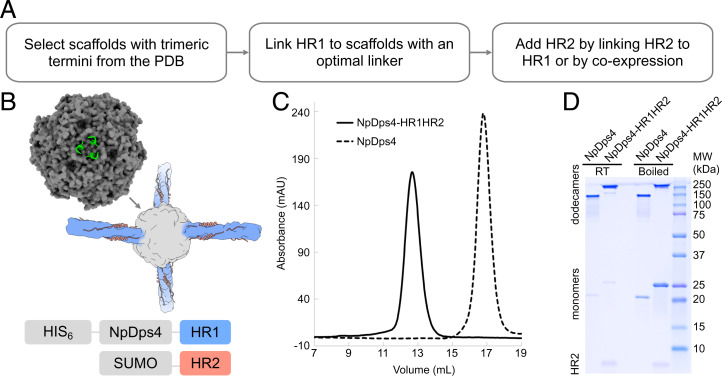
Construct design and optimization of the scaffolded HR1HR2 bundle. (*A*) Workflow of designing and optimizing the scaffolded HR1HR2 bundle. (*B*) Diagram of construct design. (*Top Left*) Crystal structure of the dodecamer Dps4 from *N. punctiforme* (NpDps4, PDB ID code 5hjf), with one of its four three-helix-bundle termini shown as a green cartoon and the rest as a gray surface. (*Middle*) Cartoon of the scaffolded HR1HR2 bundle. (*Bottom*) Diagram of the constructs for coexpression of the scaffolded HR1HR2 bundle. (*C*) Size exclusion chromatography profiles of the purified scaffolded HR1HR2 bundle and the NpDps4 scaffold alone. (*D*) SDS-PAGE gel of the purified scaffolded HR1HR2 bundle and the scaffold alone, with or without boiling. The bands above the 100 kDa marker correspond to the respective dodecamers, the bands of corresponding monomers are around the 20 kDa and 25 kDa markers, and the band for the HR2 fragment alone is below the 10 kDa marker.

We designed a scaffolded HR1HR2 bundle in three steps (Fig. 2*A*). We first surveyed the PDB for scaffold candidates with homo-trimeric termini that would be compatible with either the N or C termini of the HR1 bundle (see *SI Appendix*, *Supplementary Notes* for potential scaffolds that we considered). Such a scaffold thus needed three relatively short and tightly localized linkers between the scaffold termini and the HR1HR2 bundle, which would make the connection more rigid than that with only one linker. In addition, the scaffold candidate itself was required to be larger than 100 kDa, relatively rigid, easily produced in *Escherichia coli,* and not a membrane protein.

The protein Dps4 from *Nostoc punctiforme* (NpDps4) (PDB ID code 5hjf) (41) meets all the above criteria (Fig. 2*B*). NpDps4 is a homo-dodecameric assembly with four three-helix-bundle C termini on its four C3 rotational symmetry axes. NpDps4 has a molecular weight of 250 kDa and tetrahedral symmetry with an overall roughly spherical shape. Its structure was determined to 1.59 Å by X-ray crystallography, suggesting a rigid assembly. In addition to NpDps4, we also tested a homolog of NpDps4, Dps from *Thermosynechococcus elongatus* (TeDps, PDB ID code 2c41) (42), with the notion that proteins from thermostable species might have higher stability; however, it did not perform as well as NpDps4 (*SI Appendix*, Fig. S3*A*).

We evaluated the performance of short sequences to link the N terminus of HR1 to the C terminus of NpDps4. In these initial trials, we used a linked HR1HR2 construct [akin to using the linked construct that was used for the crystal structure of HR1HR2 (23)] in facilitating the formation of the HR1HR2 bundle (*SI Appendix*, Fig. S3). A more intense band for the presumably correctly folded dodecameric scaffold was observed by sodium dodecyl sulfate polyacrylamide gel electrophoresis (SDS-PAGE) when using the linked HR1HR2 than when using HR1 alone. A single-alanine linker between NpDps4 residue F178 and HR1 residue Y917 performed the best as judged by test expressions in *E. coli*, though several other candidates seemed to express properly as well.

We then tested different strategies of adding HR2 to the scaffolded HR1. As mentioned above, HR2 was first linked to the C terminus of HR1, akin to constructs used for X-ray crystallography (23). This approach worked sufficiently well for optimization studies of the linkers between the scaffold and HR1. However, the yield was low, presumably because the linked HR2 from one scaffold particle could interact with the HR1 from another scaffold particle, resulting in tangled multimers. Second, HR2 was added as a purified peptide to the purified scaffolded HR1. However, the yield of stable and soluble scaffolded HR1 was very low, perhaps due to the high hydrophobicity of HR1. Third, SUMO-tagged HR2 was included by coexpression with the scaffolded HR1 (Fig. 2*B* and *Materials and Methods*). This coexpression strategy proved the most effective in terms of yield and purity (Fig. 2 *C* and *D*).

The purified sample of the scaffolded HR1HR2 bundle appears as a single symmetric peak that elutes earlier than that of the scaffold itself (Fig. 2*C*), indicating an increase in size and consistent with the expectation that the particle diameter of the scaffolded HR1HR2 complex is about 320 Å and that of the NpDps4 scaffold itself is about 100 Å. By SDS-PAGE, it appears as a major dodecamer band and a minor monomer band, and many, but not all, dodecamers disassemble into monomers after boiling (Fig. 2*D*). The reason underlying this heterogeneous resistance to SDS is unclear, and we observed that the scaffold itself, as a control, had the same heterogeneous behavior. In any case, the presence of some monomeric species in the preparation did not pose a problem for single-particle cryo-EM studies. Moreover, HR2 is present in the oligomer peak as revealed by SDS-PAGE of boiled samples (Fig. 2*D*).

### Single-Particle Cryo-EM Structure Determination of the Scaffolded HR1HR2 Bundle.

**Fig. 3.**
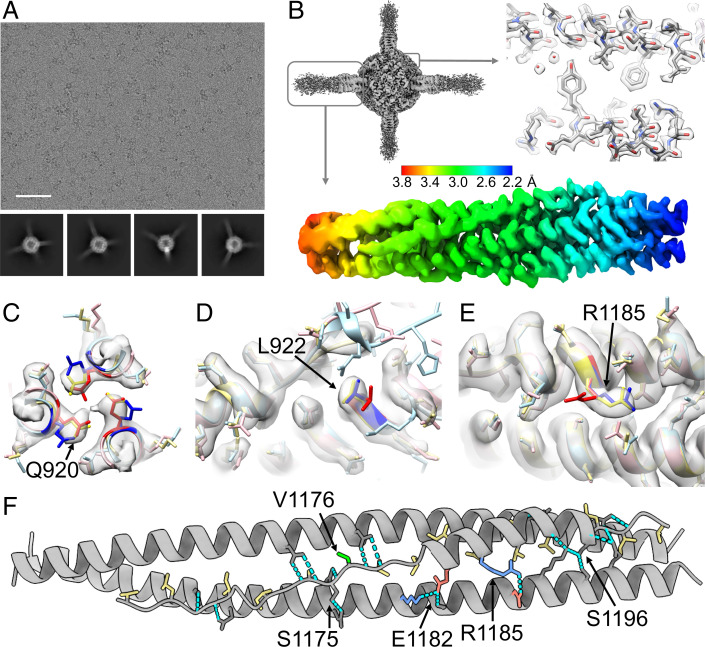
Cryo-EM studies of the scaffolded HR1HR2 complex. (*A*) Representative raw EM micrograph of the scaffolded wild-type HR1HR2 bundle and reference-free 2D class averages using a total of 2,896,745 selected particles (2D classification in cryoSPARC; *SI Appendix*, Fig. S4). The box size of the 2D class averages is 326.5 Å. (Scale bar on the raw micrograph, 500 Å.) (*B*) The globally refined map of the scaffolded HR1HR2 bundle shows the clearly resolved dodecameric NpDps4 scaffold region and four HR1HR2 complexes that are projecting from the scaffold with progressively increasing disorder. Symmetry expansion, signal subtraction, and local refinement resolve the HR1HR2 complexes, typically achieving resolutions ranging from 2.2–3.8 Å (*SI Appendix*, Fig. S4). (*C*–*E*) Comparison of the EM map (gray) of the wild-type SARS-CoV-2 HR1HR2 complex (yellow, this study) with two published crystal structures of the HR1HR2 bundle (PDB ID codes 6xlt, pink, and 6m1v, light blue), focusing on the residues with discrepant side-chain conformations between the two crystal structures. Residues Q920, L922, and R1185 of the two crystal structures are colored as red and blue, respectively, to emphasize the differences. (*F*) Analysis of key interactions between the HR1 and HR2 fragments. Residues of HR2 that are involved in hydrophobic interactions with HR1 are colored yellow. Note that the variant mutation V1176 (colored green) is not interacting with HR1. Hydrogen bonds are shown as cyan dashed lines. Salt bridges are indicated by positively charged residues (colored blue) and negatively charged residues (colored red). Only one HR2 protomer and its neighboring two HR1 protomers are shown for clarity.

The oligomeric architecture of the scaffold and the protruding HR1HR2 bundles is apparent in the raw micrographs and reference-free two-dimensional (2D) class averages (Fig. 3*A*). For data processing (*SI Appendix*, Fig. S4), we first globally refined the NpDps4 scaffolded HR1HR2 bundle to obtain a high-resolution reconstruction (up to 1.8 Å), revealing detailed densities for side chains and water molecules (Fig. 3*B*, *Right*). Despite the three-linker connection between the NpDps4 scaffold and the trimeric HR1HR2 bundle, some degree of flexibility between the scaffold and the HR1HR2 bundle exists, as evident by some disorder at the far ends of the HR1HR2 bundle in the average map of the global reconstruction (Fig. 3*B*, *Left*). Note that the disordered map at peripheral regions of a particle is commonly seen in cryo-EM and could result from a combination of factors in addition to flexibility, such as small errors in image alignment and potential exposure to the air–water interface. Regardless, global classification is a useful tool to improve the reconstructions through elimination of outliers. Next, to improve the reconstructions of the HR1HR2 bundle, we also took advantage of the fact that each scaffolded HR1HR2 particle contains four individual HR1HR2 bundles (Fig. 2*B*); we thus used the so-called symmetry expansion strategy (43, 44) to obtain four instances of the bundle from each scaffolded HR1HR2 particle (*Materials and Methods*). After symmetry expansion, signal subtraction, focused classification, and local refinement in RELION (45), we obtained a much-improved reconstruction of the HR1HR2 region (ranging from 2.2 Å to 3.8 Å resolution) where the protein backbone and many side-chain densities are clearly visible and of sufficient quality for reliable model building and automated refinement using PHENIX (46) (Fig. 3 *B*–*E*). The orientations of the final set of particles are well-distributed with a slight degree of clustering roughly along the axis of the HR1HR2 bundle (*SI Appendix*, Fig. S4*C*).

Overall, our model of the HR1HR2 bundle (PDB ID code 7rzq) is similar to the previously published cryo-EM structure of postfusion S2 at 3.0 Å resolution (PDB ID code 6xra) and to the two crystal structures (PDB ID codes 6lxt and 6m1v) of the HR1HR2 bundle at 2.9 Å and 1.5 Å resolution, respectively. Note that the HR2 fragment is synthetically linked to the HR1 fragment in both crystal structures, that HR2 is naturally linked to other parts of S2 in the postfusion EM structure of S2 (18), and that HR2 is not linked to any protein in our EM structure of the HR1HR2 bundle. Moreover, the nominally higher resolution crystal structure (PDB ID code 6m1v) excludes HR1 residues 967–988 and HR2 residues 1162–1168. Despite the overall high average real-space cross-correlation for the lower-resolution crystal structure (PDB ID code 6lxt), it has large per-residue variation, indicating poor side-chain densities or corresponding fits, especially for HR2 (*SI Appendix*, Figs. S5 and S6).

The root-mean-square deviations (RMSDs) for the backbone atoms, side-chain atoms, and all nonhydrogen atoms between our EM structure and previously determined structures are relatively small (*SI Appendix*, Table S4). However, despite the overall very similar structures, there are notable differences between the two crystal structures for the side-chain conformations of some residues such as Q920, L922, and R1185 where the electron densities from the X-ray diffraction data are poor and the corresponding models do not fit the map well (*SI Appendix*, Figs. S5 and S6). In contrast, our map and corresponding model of HR1HR2 (PDB ID code 7rzq) clearly shows a single side-chain conformation for these residues (Fig. 3 *C*–*E*).

Consistent with previously determined structures of HR1HR2 bundles from SARS-CoV, MERS-CoV, and SARS-CoV-2, our EM structure shows that the interactions between HR1 and HR2 involve an orchestrated network of hydrophobic, hydrogen bonding, and ionic interactions (Fig. 3*F*). Most of the hydrophobic residues of HR2 are involved in hydrophobic interactions with HR1, with only one exception—V1176, which is completely solvent-exposed. Another notable feature of the interactions between HR1 and HR2 is the abundance of hydrogen bonding between HR1 side chains and the HR2 backbone. Thirteen out of the 19 hydrogen bonds between HR1 and HR2 are formed between HR1 side chains and the HR2 backbone, and only six remaining hydrogen bonds involve the side chains of HR2, including S1175 and S1196 (Fig. 3*F*). In addition, two salt bridges, D936/R1185 and K947/E1182, are formed.

### The Mutations of HR1HR2 Bundle Do Not Alter the Overall Architecture.

**Fig. 4.**
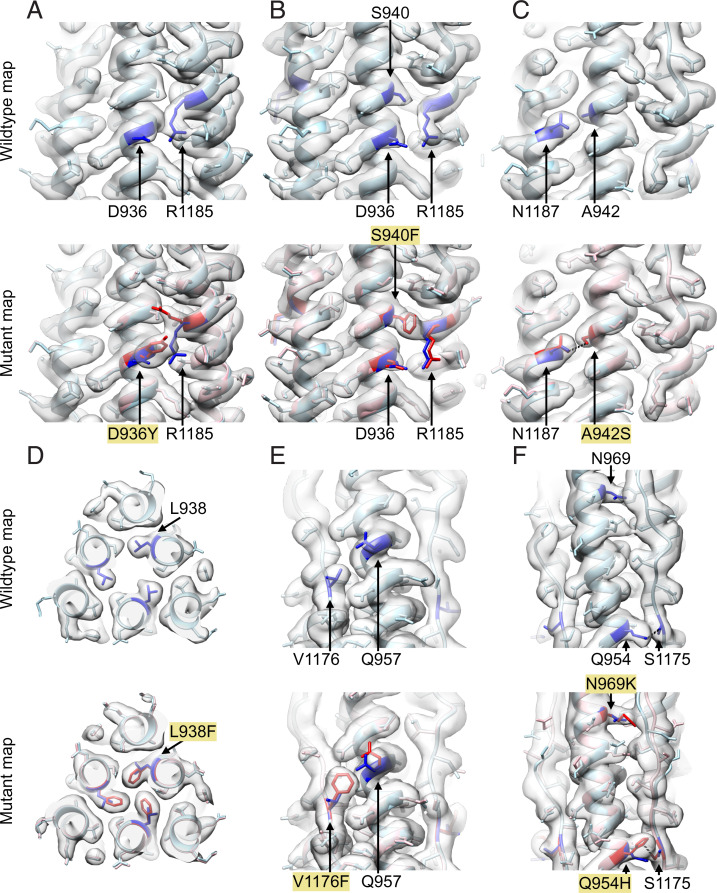
Local variation and global conservation of the structures of the studied mutants. (*A*–*F*) The EM structures of the HR1HR2 D936Y, S940F, A942S, L938F, and V1176F single mutants and the Omicron HR1HR2 triple mutant are shown in *A* through *F*, respectively. In each panel, the wild-type structure (light blue) and wild-type map are shown on the top, and the wild-type and mutant (pink) structures, and the mutant map are shown on the bottom. The mutated residues and affected residues are colored blue for the wild-type structure and red for the mutant structures. The hydrogen bonds between S942 and N1187 (*C*), Q954 and S1175 (*F*), and H954 and S1175 (*F*) are shown as black dashed lines.

We determined single-particle cryo-EM structures of the five selected single mutants and the Omicron triple mutant of HR1HR2 (Fig. 1*C*) using the same molecular scaffold (NpDps4) and data processing workflow as for the EM structure of the wild-type HR1HR2 bundle. Our approach yielded near-atomic resolution structures of the mutant HR1HR2 bundles (Fig. 4 and *SI Appendix*, Tables S2 and S3). The side-chain density for each mutated site generally reflects the corresponding mutation (Fig. 4).

The structures reveal local structural changes around some of the single mutations. For example, the D936Y mutation (Fig. 4*A*) causes a large conformational change of the side chain of HR2 residue R1185, while the S940F mutation (Fig. 4*B*) results in disappearance of side-chain density for R1185, suggesting disorder of this side chain. Thus, in both cases, the salt bridge between D936 and R1185 is disrupted by the respective variant mutants. This salt bridge disruption is associated with decreased cell–cell fusion activities for the D936Y and S940F mutants (65.8% and 24.6%, respectively, relative to that of the wild type [Fig. 1*C*]). In marked contrast, the A942S mutation (Fig. 4*C*) leads to one additional hydrogen bond between HR1 residue S942 and HR2 residue N1187, along with an approximately threefold increase of fusion activity (Fig. 1*C*). The L938F mutation fills a hole in the HR1 bundle (Fig. 4*D*), but it decreases the fusion activity to 67% of wild type (Fig. 1*C*). The V1176F mutation (Fig. 4*E*), similar to the wild type, remains solvent-exposed but slightly displaces Q957 on HR1 and also decreases fusion activity (Fig. 1*C*).

In the Omicron HR1HR2 triple-mutant structure (Q954H, N969K, L981F) (Fig. 4*F*), the backbone of HR2 is slightly displaced, likely by the N969K mutation. The L981F mutation may also contribute to the small shift of the HR2 backbone, but its side-chain density is not well-resolved, perhaps since it is located furthest from the center of the scaffold. The slight displacement seems to be unrelated to the Q954H mutation since the hydrogen bond between the HR1 residue 954 and the HR2 residue S1175 is maintained, before and after the Q954H mutation. Overall, none of the five single and the Omicron triple mutations dramatically altered the architecture of the HR1HR2 bundle, as indicated by the small RMSDs between the wild-type and the mutant structures (*SI Appendix*, Table S4). Although the Omicron triple mutant shows a slightly larger deviation from the wild-type structure than that of the five selected single mutants, its three mutation sites are all found within the nonhelical region of HR2 and relatively far from the six-helix-bundle region.

## Discussion

In this work we determined structures of the postfusion HR1HR2 bundle of SARS-CoV-2 and variant-based mutants by single-particle cryo-EM. Our work also serves as an example of scaffolding small proteins by multiple connections for high-resolution structure determination. Conformational heterogeneity is notoriously detrimental to high-resolution reconstruction in cryo-EM. Here we show that connecting a relatively small protein complex to an appropriately symmetric molecular scaffold by multiple linkers enables near-atomic-resolution structure determination downstream using signal subtraction, symmetry expansion, and focused classification methods (Fig. 3 and *SI Appendix*, Fig. S4). In principle, this approach could be applied to studying other relatively small protein complexes, although optimization of the choice of molecular scaffold and synthetic linkers to the target protein complex may be required. We also note that the scaffold approach developed in this study can be directly used to determine the structures of potential HR2-based inhibitors bound to HR1, providing a powerful tool for inhibitor screening and design.

Overall, our wild-type structure is similar to previously determined structures of the HR1HR2 bundle. However, some side chains that were assigned to different positions in the prior structures are clarified in our EM map (Fig. 3 *C*–*E*). The maps of the five selected variant-based mutations in the HR1HR2 bundle show clear densities for the mutated residues. We observed various effects on neighboring residues for the D936Y, S940F, V1176F, A942S, and the Omicron triple mutant (Fig. 4). These structural effects correlate with changes in fusion activities for these mutants (Fig. 1*C*)—the D936Y and S940F mutations result in a loss of a salt bridge along with a decrease in fusion activity, the V1176F mutation on HR2 slightly displaces Q957 on HR1 along with a decrease in fusion activity, and the A942S mutation introduces an additional hydrogen bond along with an increase in fusion activity. However, the observed decreases in fusion activities for the L938F mutation cannot be as easily explained by the observed structure—the mutant phenylalanine side chain is well determined in the map and it does not produce an apparent change of the surrounding atoms. It is possible, or even likely, that the mutations affect the membrane fusion mechanism in a way that is not related to the postfusion state; for example, this mutation as well as the others could affect the stability of the prefusion spike, or the transition from the prefusion state to the extended intermediate state of the S2 (14). Furthermore, these mutations could also have epistatic effects in the context of other mutations outside of the HR1HR2 region. Thus far it has been difficult to image extended intermediate states of viral proteins at high resolution, although a cryo-electron tomography study (17) supports the existence of such an intermediate state. Future studies of SARS-CoV-2 and other coronaviruses will be required to characterize intermediate states of S2.

Despite differences in detailed side-chain positions and slight displacement of HR2 backbone in its nonhelical region, the overall structure of the HR1HR2 bundle is remarkably similar for both wild-type and mutant HR1HR2 complexes, including Omicron (Fig. 4). This suggests that inhibitors aimed at disrupting the postfusion HR1HR2 bundle (23–25) might be broadly efficacious, even among variants. Such inhibitors could be used as alternatives or complements to therapeutics targeting receptor binding, replication, and release (47). More generally, considering remarkable structural conservation (*SI Appendix*, Fig. S1), similar strategies are also applicable to other coronaviruses including SARS-CoV (PDB ID code 2bez) (48), MERS-CoV (PDB ID code 4njl) (49), and the human coronavirus 229E (PDB ID code 5yl9) (50), as well as other enveloped viruses—extant and emerging—that rely on the class I fusion proteins for infection.

## Materials and Methods

### Molecular Cloning.

The DNA sequences of scaffolds, HR1, HR2, and the SUMO tag were codon-optimized for *E. coli* by the GeneOptimizer algorithm (51), synthesized by the Integrated DNA Technologies company, and cloned into the Duet expression system (Novagen) using Gibson assembly (52) (see *SI Appendix*, *Supplementary Notes* for details). The final optimized constructs for the wild-type HR1 and HR2 fragments contain residues 917–988 and 1162–1201 of the spike protein, respectively. The constructs for the cell–cell fusion assay (18) were kindly provided by Bing Chen, Harvard Medical School, Boston, including the full-length wild-type S, the full-length wild-type ACE2, and the α-fragment and ω-fragment of *E. coli* β-galactosidase, all cloned in the pVRC8400 vector (53). Point mutations and short insertions were introduced using the Q5 site-directed mutagenesis kit with primers designed by the NEBaseChanger tool (New England Biolabs). The construct for the enhanced SUMO protease with a C-terminal deca-histidine tag was purchased from Addgene (pCDB302, Addgene ID 113673) (54). The NEB Turbo competent *E. coli* cells were used to replicate the constructs.

### Cell–Cell Fusion Assay.

The cell–cell fusion assay that measures the fusion activity of SARS-CoV-2 S based on the α-complementation of *E. coli* β-galactosidase (18) was performed with modifications as described below. The S-expressing cells were made by cotransfecting Expi293F (Thermo Fisher) cells with the full-length S construct and the α-fragment construct of *E. coli* β-galactosidase, for the wild-type S and each S variant. The transfection was performed with precisely 12.5 μg of each of the two constructs and 125 μg polyethyleneimine, followed by 24-h incubation at 37 °C. The ACE2-expressing cells were made in the same way with the full-length ACE2 construct and the ω-fragment construct of *E. coli* β-galactosidase. The cell–cell fusion was initiated by mixing 50 μL S-expressing cells at a density of 2 × 10^6^ cells per milliliter and 50 μL ACE2-expressing cells at the same density in a 96-well plate, followed by 2-h incubation at 37 °C. Following the Gal-Screen reporter system, 100 μL β-galactosidase substrate was added to each cell mixture, followed by 1-h incubation at 37 °C in the dark before recording luminescence using a Tecan Infinite M1000. The luminescence reading of each S variant was normalized to that of wild-type S.

### Protein Expression and Purification.

To express the SUMO protease, the scaffold alone, and the scaffolded HR1HR2 complex, or to coexpress the scaffolded HR1 and SUMO-HR2, the BL21(DE3), competent *E. coli* cells transformed with the corresponding plasmids were grown in autoinducing lysogeny broth medium (55) at 37 °C for 4 h to reach saturation and then at 25 °C overnight to induce protein expression. Cells were harvested by centrifugation and stored at −80 °C for further use.

The harvested cell paste was resuspended in lysis buffer [50 mM sodium phosphate, pH 8.0, 300 mM NaCl, 20 mM imidazole, 0.5 mM Tris(2-carboxyethyl)phosphine (TCEP), and 0.5% Triton X-100] supplemented with lysozyme, DNase, and ethylenediaminetetraacetic acid (EDTA)–free protease inhibitors, lysed by sonication, and centrifuged at 39,000 × *g* at 4 °C for 30 min. The supernatant was incubated with Ni^2+^-NTA resin, preequilibrated with the lysis buffer, at 4 °C for 1 h. The Ni^2+^-NTA resin was washed sequentially with wash buffer 1 (50 mM sodium phosphate, pH 8.0, 300 mM NaCl, 30 mM imidazole, 0.5 mM TCEP, and 0.5% Triton X-100) and wash buffer 2 (50 mM sodium phosphate, pH 8.0, 300 mM NaCl, 30 mM imidazole, and 0.5 mM TCEP) and then eluted with elution buffer (50 mM sodium phosphate, pH 8.0, 300 mM NaCl, 300 mM imidazole, and 0.5 mM TCEP). The eluted sample was then cleaned by a Superose 6 Increase 10/300 GL column in SEC buffer (25 mM Hepes-Na, pH 7.4, 150 mM NaCl, 0.5 mM EDTA, and 0.5 mM TCEP). For the samples with a SUMO tag on HR2, the pooled fractions were cleaved by the enhanced SUMO protease overnight at 4 °C and further cleaned by a Superose 6 Increase 10/300 GL column in the SEC buffer. Good fractions were pooled based on the purity in SDS-PAGE, concentrated with a 100 kDa cutoff concentrator to about 50 μM dodecamer concentration, aliquoted, flash-frozen in liquid nitrogen, and stored at −80 °C.

### Cryo-EM Sample Preparation and Data Collection.

The samples were diluted to 20 μM in the SEC buffer supplemented with 0.05% Nonidet P-40 before cryo-EM sample preparation. Using a Vitrobot Mark IV (Thermo Fisher Scientific), 3.5 μL sample solution at 20 μM was applied to a Quantifoil 2/1 holey carbon grid at 10 °C and 95% relative humidity and vitrified by plunge freezing after removing excess liquid by blotting for 2 s with a blotting force of 1. The grids were imaged using a Titan Krios electron microscope (Thermo Fisher Scientific) equipped with a K3 camera (Gatan) using the Serial-EM automation software (56). The nominal magnification was 130,000× and the pixel size was 0.3265 Å. The dose rate was around 20 electrons per physical pixel per second. At each stage position, a group of nine holes was imaged using the multiple record setup (56, 57), and each hole contained six imaging spots. At each imaging spot, a 40-frame movie stack was collected with a total exposure time of 1.012 s. The data collection speed was about 9,000 movie stacks per day. More details for data collection are summarized in *SI Appendix*, Table S2.

### Cryo-EM Data Processing.

All datasets were processed with the RELION-3 package (45) except when explicitly noted (*SI Appendix*, Fig. S4*A*). The raw movie stacks were aligned, dose-weighted, and summed using RELION’s implementation of the MotionCor2 program (58), with five patches in the X direction and three patches in the Y direction and a binning factor 2, yielding a pixel size of 0.653 Å. The CTF parameters of the summed micrographs were estimated using Gctf with equi-phase averaging (59). Good micrographs were selected based on the following criteria: rlnCtfMaxResolution < 5 Å, rlnCtfAstigmatism < 1,000 Å, 3,000 Å < rlnDefocusU < 20,000 Å. Particles were picked using the e2boxer.py in EMAN2 (60) using a convolutional neural network (61). The calculated particle coordinates were imported into RELION for the first round of cleaning up and recentering based on the dodecameric NpDps4 scaffold (Fig. 2*B*). The particles were extracted with a binning factor of 4, yielding a pixel size of 2.612 Å, and with a diameter background circle of 100 Å which only covers the scaffold region, and with a box size of 48 pixels. The extracted particles were subjected to one round of reference-free 2D classification and one round of 3D classification with T symmetry and a 60 Å low-pass-filtered initial model that was generated in RELION using the particles after the 2D classification. Based on the good 3D classes, the particles were recentered and reextracted with a binning factor of 1.25 (effective pixel size: 0.816 Å), a box size of 400 pixels, and a background circle of 320 Å diameter covering the entire scaffolded HR1HR2 particle.

The recentered and reextracted particles were imported to cryoSPARC to rapidly remove particle pairs that are either overlapping or too close to each other by one round of reference-free 2D classification, and to align the scaffold region as accurately as possible by one round of “homogeneous refinement” with tetrahedral symmetry, the default dynamic masking, and on-the-fly CTF refinement including optimization of per-particle defocus and per–exposure group CTF parameters, but not of anisotropic magnification or CTF astigmatism. We found that cryoSPARC (62) (with default settings, i.e., without optimizing the refinement parameters at an expert level) yielded the best resolution for the NpDps4 scaffold region. Perhaps the dynamic masking in cryoSPARC allowed the iterative alignment focusing on the more rigid scaffold and ignoring the more flexible HR1HR2 bundle region. The refined particles were imported back to RELION for Bayesian polishing, recentering, and reextracting the polished particles from the raw movie stacks with a binning factor of 2.5 (effective pixel size: 0.816 Å) and a box size of 560 pixels. The polished particles were imported to cryoSPARC and subjected to a final round of “homogeneous refinement” with the same setting.

The polished and cryoSPARC-refined particles were imported to RELION for tetrahedral symmetry expansion (43, 44), yielding 12 copies per original particle. Since each original scaffolded HR1HR2 particle only contains four copies of a HR1HR2 bundle, the extra eight copies in the tetrahedral symmetry expanded particle set were discarded by a Python script. This process selects one from the three copies around each of the four C3 rotational axes in the tetrahedral symmetry. In other words, each protruding HR1HR2 bundle density (together with the attached scaffold) was treated as an individual “particle.” The signal outside a spherical mask (generated in RELION) (*SI Appendix*, Fig. S4*A*) covering one HR1HR2 bundle and part of the scaffold was subtracted from the particles, yielding particles containing only the masked region. The subtracted particles were recentered at the voxel (0, 0, −95) in the original particle, a point near the center of the mask for subtraction and slightly toward the scaffold region, with a new box size of 256 pixels. The subtracted particles were subjected to one round of skip-align (without alignment) 3D classification to select the particles with good densities in the HR1HR2 bundle, and one round of 3D autorefinement with local angular searches and with symmetry relaxation from C3 to C1 to orientate the subtracted particles to a consensus pose, using the best class as the initial model after low-pass filtering to 7 Å. One more round of skip-align 3D classification, with another spherical mask (generated in RELION) (*SI Appendix*, Fig. S4*A*) covering only the HR1HR2 bundle, yielded an apparently best class, which was subjected to another round of 3D autorefinement with local angular searches, using the corresponding class as the initial model after low-pass filtering to 7 Å. Additional rounds of CTF refinement did not improve the resolution. The refined maps were automatically sharpened by DeepEMhancer, a Convolutional Neural Networks–based postprocessing program (63).

### Molecular Modeling.

The PDB ID code 6lxt was used as the template for real-space refinement (minimization_global, local_grid_search, adp) in PHENIX (46). Point mutations were made in UCSF Chimera (64). The fitting of side chains in the map was manually inspected and corrected in Coot (65).

### Figure Preparation.

The figures of PDB structures and maps were made in PyMOL (The PyMOL Molecular Graphics System, Version 2.5, Schrödinger, LLC), UCSF Chimera (64), or ChimeraX (66).

## Supplementary Material

Supplementary File

## Data Availability

The EM maps and models from this work have been deposited in EMDB (Electron Microscopy Data Bank) and PDB with the following accession IDs: HR1HR2 wild type: EMDB 24774, PDB 7rzq; HR1HR2 D936Y: EMDB 24775, PDB 7rzr; HR1HR2 L938F: EMDB 24776, PDB 7rzs; HR1HR2 S940F: EMDB 24777, PDB 7rzt; HR1HR2 A942S: EMDB 24778, PDB 7rzu; HR1HR2 V1176F: EMDB 24779, PDB 7rzv; and HR1HR2 Omicron: EMDB 25912, PDB 7tik.
