# Supplementary material for: Structural conservation among variants of the SARS-CoV-2 spike postfusion bundle
Source: Proc Natl Acad Sci U S A. 2022 Apr 1;119(16):e2119467119. doi: 10.1073/pnas.2119467119 (PMC9169775; doi:10.1073/pnas.2119467119)
Supplement: Supplementary File [file pnas.2119467119.sapp.pdf]

PNAS
www.pnas.org

**Supplementary Information for**

Structural conservation among variants of the SARS-CoV-2 spike post-fusion bundle

Kailu Yang, Chuchu Wang, K. Ian White, Richard A. Pfuetzner, Luis Esquivies, Axel T. Brunger

Corresponding author: Axel T. Brunger
Email: brunger@stanford.edu

**This PDF file includes:**

Figures S1 to S6
Tables S1 to S4
Supplementary Notes
SI References

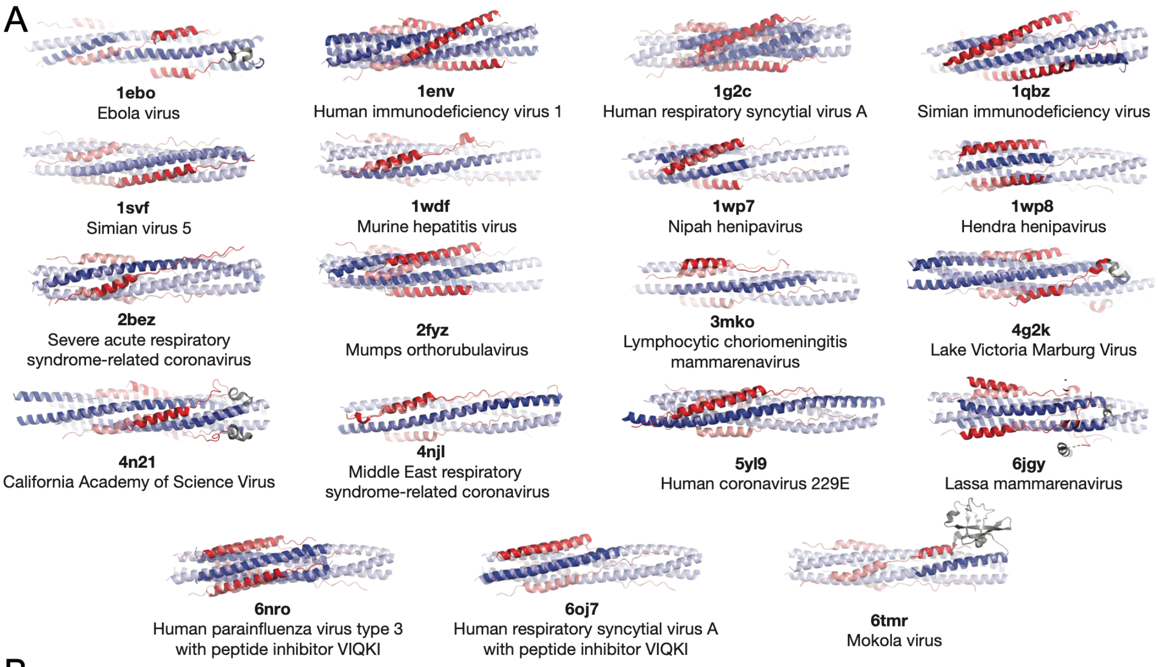


B

```
Conservation:                             75  9              7  9  5  97  6              5  8  58  6
6lxt          --------TQNVLYENQKLIANQF--NSAIGKI--------------QDSLSSTASALGKLQD-----------VVNQNAQALNTLVKQ---LSS
1ebo          ----------QIEDKIEEILSKIYHIENEIARI--------------KKLIGEADGLIEGLRQ-----------LANETTQALQLFLRATTELRT
1env          ----------QIEDKIEEILSKIYHIENEIARI--------------KKLIGEARQLLSGIVQ-----------QQNNLLRAIEA-QQHLLQLTV
1g2c          -------------------------LEGEVNKI--------------KSALLSTNKAVVSLSN-----------GVSVLTSKVLDLKNY------
1qbz          -------------QSRTLLAGI---VQQQQQL--------------LDVVKRQQELLRLTVW-----------GTKNLQTRVTAIEKY------
1svf          -------TAAVALVKANEN-------AAAILNL--------------KNAIQKTNAAVADVVQ-----------ATQSLGTAVQAVQDH------
1wdf          ---------------QKMIASAF--NNALGAI--------------QDGFDATNSALGKIQS-----------VVNANAEALNNLLNQ---LSN
1wp7          ----------------------------NINKL--------------KSSIESTNEAVVKLQE-----------TAEKTVYVLTALQ--------
1wp8          ----------------------------NINKL--------------KSSIESTNEAVVKLQE-----------TAEKTVYVLTALQ--------
2bez          ----------NVLYENQKQIANQF--NKAISQI--------------QESLTTTSTALGKLQD-----------VVNQNAQALNTLVKQ---LSS
2fyz          --------SAVSLVQAQTN-------ARAIAAM--------------KNSIQATNRAVFEVKE-----------GTQRLAIAVQAIQDH------
3mko          ----------------------------EEFSDM--------------LRLIDYNKAALSKFKQDVESALHVFKTTVNSLISDQLLMRNH------
4g2k          --------MKQIEDKIEEILSKIYHIENEIARI--------------KKLIGN---LVSRLRR-----------LANQTAKSLELLLRVTTEERT
4n21          ENLYFQGNMKQIEDKIEEILSKIYHIENEIARI--------------KKLIGA---IASKIIK-----------TANYTTNALFLLNKEESEIRD
4njl          -------ITQQVLSENQKLIANKF--NQALGAM--------------QTGFTTTNEAFQKVQD-----------AVNNNAQALSKLASE---LSN
5yl9          ----------DVLQENQKILAASF--NKAMTNIVDAFTGVNDAITQTSQALQTVATALNKIQD-----------VVNQQGNSLNHLTSQ---LRQ
6jgy          --------------------------DEEFSDM--------------LRLFDFNKQAIQRLKAEAQMSIQLINKAVNALINDQLIMKNH------
6nro          --------------------------RSDIEKL--------------KEAIRDTNKAVQSVQS-----------SIGNLIVAIKSVQDY------
6oj7          H------------------------LEGEVNKI--------------KSALLSTNKAVVSLSN-----------GVSVLTSKVLDLKNY------
6tmr          ----------DRLDEIEHLIV--------------------------EDIIKKREECLDTLET-----------ILMSQSVS-------------
Consensus_aa: ...........................p.h.ph..............pp.l..ppphl.pl.p............hp....sl..l.p.......
Consensus_ss:                hhhhhh     hhhhhhh              hhhhhhhhhhhhhhhh           hhhhhhhhhhhhhhh

Conservation:     7                                            6    7       6  9             6      8  7
6lxt          NFGAIS-SVLNDILSRL--DKVE--        -----VDLGDISGINASVVNI----QKEIDRL-------NEVAKNLNESLIDLQE-------
1ebo          F-SILNRKAIDFLLQRW--------        ---TCHIL--GPDCRIEPHDW----TKNITDK-------IDQIIHDFVDK------------
1env          W-GIKQLQARILAVERYLK------        ----------WMEWDREINNYTSLIHSLIEES-------QNQQEKNEQELLELDK-------
1g2c          ----IDKQ-LLPIVNK---------        --PLVF---PSDEFDASISQV----NEKINQS-------LAFIRKSDELLHNVNAG------
1qbz          ----LKDQ---AQLNAWG-------        --TPKWNNETWQEWERKVDFLEENITALLEEA-------QIQQEKNMYELQKL---------
1svf          ----INSV-VSPAITAANY------        --QILSI--DPLDISQNLAAV----NKSLSDA-------LQHLAQSDTYLSAI---------
1wdf          RFGAI--------------------        ---DLSL--DFEKLNVTLLDL----TYEMNRI-------QDAIKKLNESYINL---------
1wp7          -------------------------        ------------DISSQISSM----NQSLQQS-------KDYIKEAQRLLDTV---------
1wp8          -------------------------        ------------DISSQISSM----NQSLQQS-------KDYIKEAQKILDTV---------
2bez          NFGAIS-SVLNDILSRL--DKVEAE        TSPDVD-LGDISGINASVVNI----QKEIDRL-------NEVAKNLNESLIDLQ--------
2fyz          ----INTI-MNTQ------------        ------------DISTELSKV----NASLQNT-------VKYIKESNHQLQSVIV-------
3mko          ----LRDLMGVPYCNYSKFWYLEHA        --PKCWLV--TNGSYLNETHF----SDQIEQE-------ADNMITEMLR-------------
4g2k          F-SLINRHAIDFLLTRW--------        ---TCKVL--GPDCSIGIEDL----SRNISEQ-------IDQIKKDEQK-------------
4n21          H-VVEHELALNYLLAHQ--------        ---LCNVVKGP-MCSSDIDDF----SKNVSDM-------IDKVHEEMKKFYHE---------
4njl          TFGAIS-ASIGDIIQRL--DVLEQ-        SIPNFG---SLTQINTTLLDL----TYEMLSL-------QQVVKALNESYIDLKELGN----
5yl9          NFQAIS-SSIQAIYDRL--DTI---        -VPDLV----VEQYNQTILNL----TSEISTLENKSAELNYTVQKLQTLIDNINSTLVDLKW
6jgy          ----LRDIMGIPYCNYSKYWYLNH-        --PKCWLV--SNGSYLNETHF----SDDIEQQ-------ADNMITEMLQKE-----------
6nro          ----VNKE-IVPSIAR---------        -------ALDPIDISIVLNKI----KSQLEES-------KEWIRRSNKILDSI---------
6oj7          ----IDKQ-LLPI------------        -------ALDPIDFSIVLNKI----KSQLEES-------KEWIRRSNKILDSI---------
6tmr          -------------------------        -----------PDGQILIPEM---QSEQLKQH-------MDLLKAAVFPLRHPLI-------
Consensus_aa: ....lp...................        .............hp.pl.ph....p.plpp........p.h.p....h.p...........
Consensus_ss:     hhhh hhhhh                                h hhhhhh    hhhhhhh       hhhhhhhhhhhh
```

**Fig. S1.** Survey of X-ray crystal structures of viral post-fusion bundles from the PDB. (A) Presented structures (1-18) were identified based on structural homology to the SARS-CoV-2

post-fusion HR1HR2 bundle (PDB 6lxt) (19). In cases where only one HR1HR2 chain was present in an asymmetric unit, the bundle was generated by the appropriate symmetry operations if possible. Structural alignment was performed using PyMOL (The PyMOL Molecular Graphics System, Version 2.5, Schrödinger, LLC.) or UCSF Chimera (20); HR1 was used as the primary alignment target. HR1 is shown in blue, and HR2 in red. For each structure, PDB 6lxt is shown with transparency for reference. Additional secondary structure elements are shown in grey. The fundamental 6HB structure of the post-fusion bundle is remarkably well conserved, despite extremely divergent sequence identity between many of these viruses. (B) Sequence alignment of HR1 and HR2 by PROMALS3D (21). HR1 and HR2 sequences are shown sequentially and separated by space. In the alignment, the first line shows conservation indices for positions with a conservation index above 5. The conservation indices are integers between 0 and 9, with 9 corresponding the highest conservation. The last two lines show consensus amino acid sequence (*Consensus_aa*) and consensus predicted secondary structures (*Consensus_ss*). Representative sequences have magenta names and they are colored according to predicted secondary structures (red: alpha-helix, blue: beta-strand). Consensus predicted secondary structure symbols: alpha-helix: *h*. Consensus amino acid symbols are: aliphatic (I, V, L): l; hydrophobic (W, F, Y, M, L, I, V, A, C, T, H): h; polar residues (D, E, H, K, N, Q, R, S, T): *p*; small (A, G, C, S, V, N, D, T, P): *s*; bulky residues (E, F, I, K, L, M, Q, R, W, Y): *b*.

```
HR1
wildtype   917-YENQ KLIANQFNSA IGKIQDSLSS TASALGKLQD VVNQNAQALN TLVKQLSSNF GAISSVLNDI LSRLDKVE-988
D936Y      917-YENQ KLIANQFNSA IGKIQYSLSS TASALGKLQD VVNQNAQALN TLVKQLSSNF GAISSVLNDI LSRLDKVE-988
L938Y      917-YENQ KLIANQFNSA IGKIQDSFSS TASALGKLQD VVNQNAQALN TLVKQLSSNF GAISSVLNDI LSRLDKVE-988
S940F      917-YENQ KLIANQFNSA IGKIQDSLSF TASALGKLQD VVNQNAQALN TLVKQLSSNF GAISSVLNDI LSRLDKVE-988
A942S      917-YENQ KLIANQFNSA IGKIQDSLSS TSSALGKLQD VVNQNAQALN TLVKQLSSNF GAISSVLNDI LSRLDKVE-988
V1176F     917-YENQ KLIANQFNSA IGKIQDSLSS TASALGKLQD VVNQNAQALN TLVKQLSSNF GAISSVLNDI LSRLDKVE-988
Omicron    917-YENQ KLIANQFNSA IGKIQDSLSS TASALGKLQD VVNHNAQALN TLVKQLSSKF GAISSVLNDI FSRLDKVE-988

HR2
wildtype   1162-PDVDLGDIS GINASVVNIQ KEIDRLNEVA KNLNESLIDL Q-1201
D936Y      1162-PDVDLGDIS GINASVVNIQ KEIDRLNEVA KNLNESLIDL Q-1201
L938Y      1162-PDVDLGDIS GINASVVNIQ KEIDRLNEVA KNLNESLIDL Q-1201
S940F      1162-PDVDLGDIS GINASVVNIQ KEIDRLNEVA KNLNESLIDL Q-1201
A942S      1162-PDVDLGDIS GINASVVNIQ KEIDRLNEVA KNLNESLIDL Q-1201
V1176F     1162-PDVDLGDIS GINASFVNIQ KEIDRLNEVA KNLNESLIDL Q-1201
Omicron    1162-PDVDLGDIS GINASVVNIQ KEIDRLNEVA KNLNESLIDL Q-1201
```

**Fig. S2.** Sequence alignment of the wildtype, the five single mutant, and the Omicron triple mutant HR1HR2. The residue number, based on the wildtype SARS-CoV-2, is labeled for N and C terminal residues of HR1 and HR2. Mutated residues are indicated by the bold underscored font.

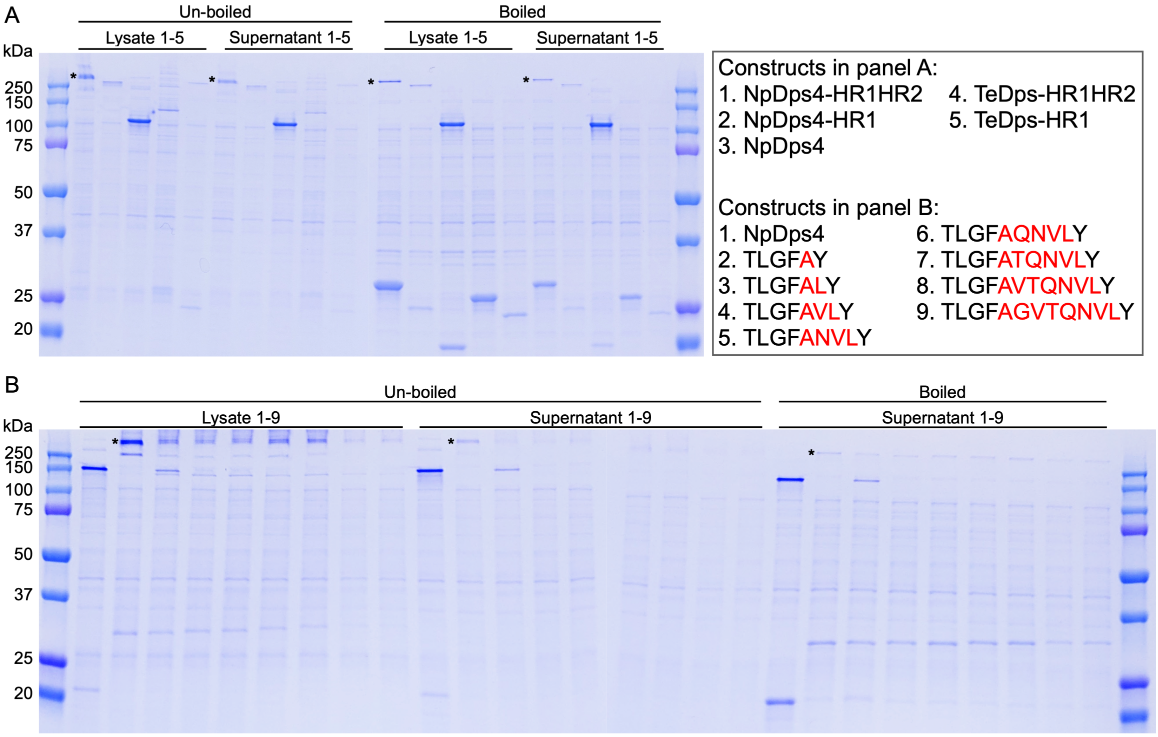


**Fig. S3.** Test expression of various constructs of scaffolded HR1HR2 bundle. (A) SDS gel of test expressions in *E. coli* to compare the NpDps4 and TeDps scaffolds, and to assess the effect of linking HR2 to HR1 (the sequence SGGRGG is used to link the HR1 residue E988 and HR2 residue P1162). Note, however, that in the final protocol, co-expression was used rather than linkage between HR1 and HR2 (**Fig. 2B-D**). An alanine linker is used to link HR1 residue Y917 and residue F178 of the NpDps4 scaffold. The HR1 residue Q918 is directly linked to the residue K153 of the scaffold TeDps. (B) SDS gel of test expressions in *E. coli* to assess the effects of various linkers between the NpDps4 scaffold and HR1 with increasing number of residues. The sequences of the linkers between residue T175 of scaffold NpDps4 and HR1 residue Y917 are shown, with the red color indicating the differences among linkers. The star sign indicates the dodecamer band for the optimal construct.

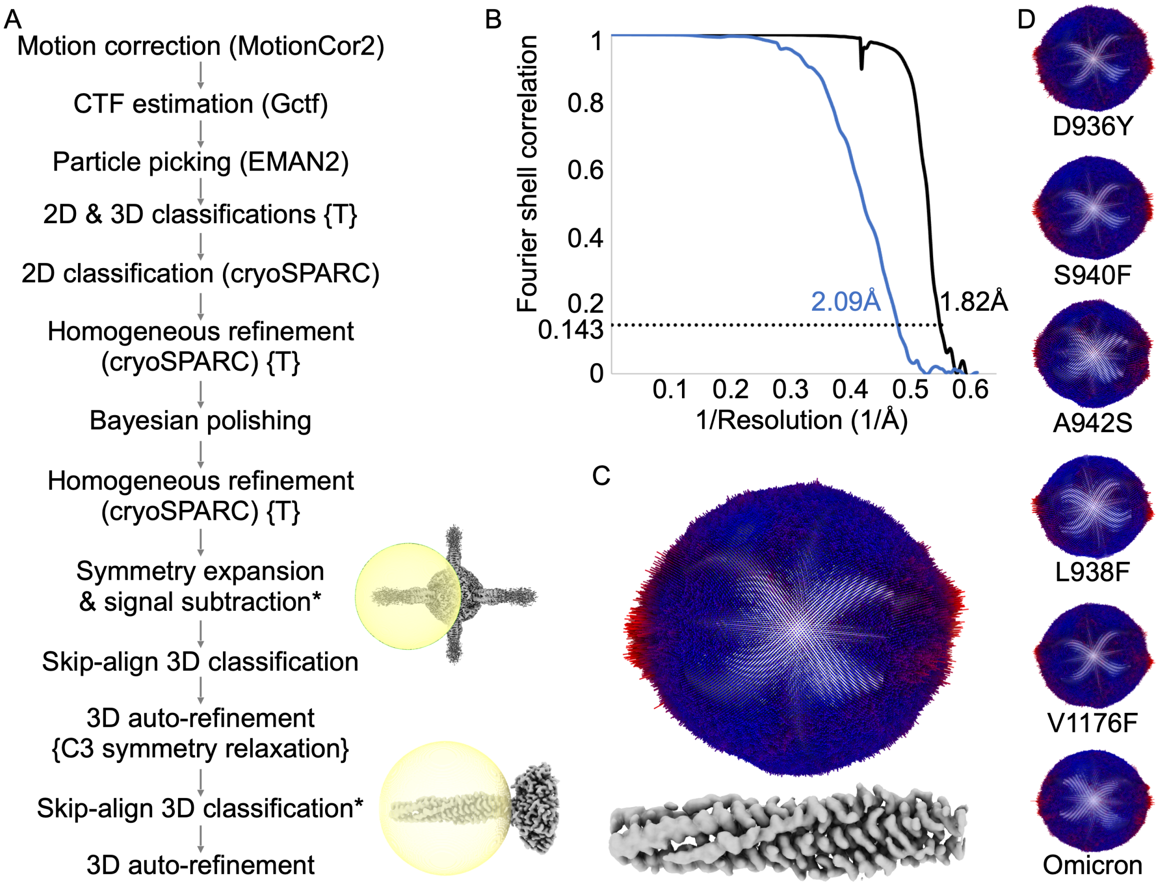


**Fig. S4.** Cryo-EM structure determination. (A) Workflow of cryo-EM data processing. RELION was used for each of the steps unless another program is indicated in parenthesis. Symmetry was imposed or relaxed as indicated in curly brackets. The steps that used a manually generated mask (rather than the default spherical mask in RELION or the default dynamic masks in cryoSPARC) are indicated with a star sign and an image showing the manually generated mask (yellow) and an average map (gray). (B) Fourier shell correlations (FSC) of the final refinement step with cryoSPARC (second "homogeneous refinement" using cryoSPARC, black curve) and the final local refinement with RELION (last 3D auto-refinement, blue curve). (C) Distribution of the particles' orientations in the final reconstruction of the wildtype dataset of the scaffolded SARS-CoV-2 HR1HR2 bundle. The length of each bar is proportional to the number of particles oriented in the direction of that bar. The bars are also colored based on the length, with red meaning more particles and blue meaning less particles. (D) Orientation distributions of the SARS-CoV-2 mutant datasets, depicted in the same orientation and color scheme as (C).

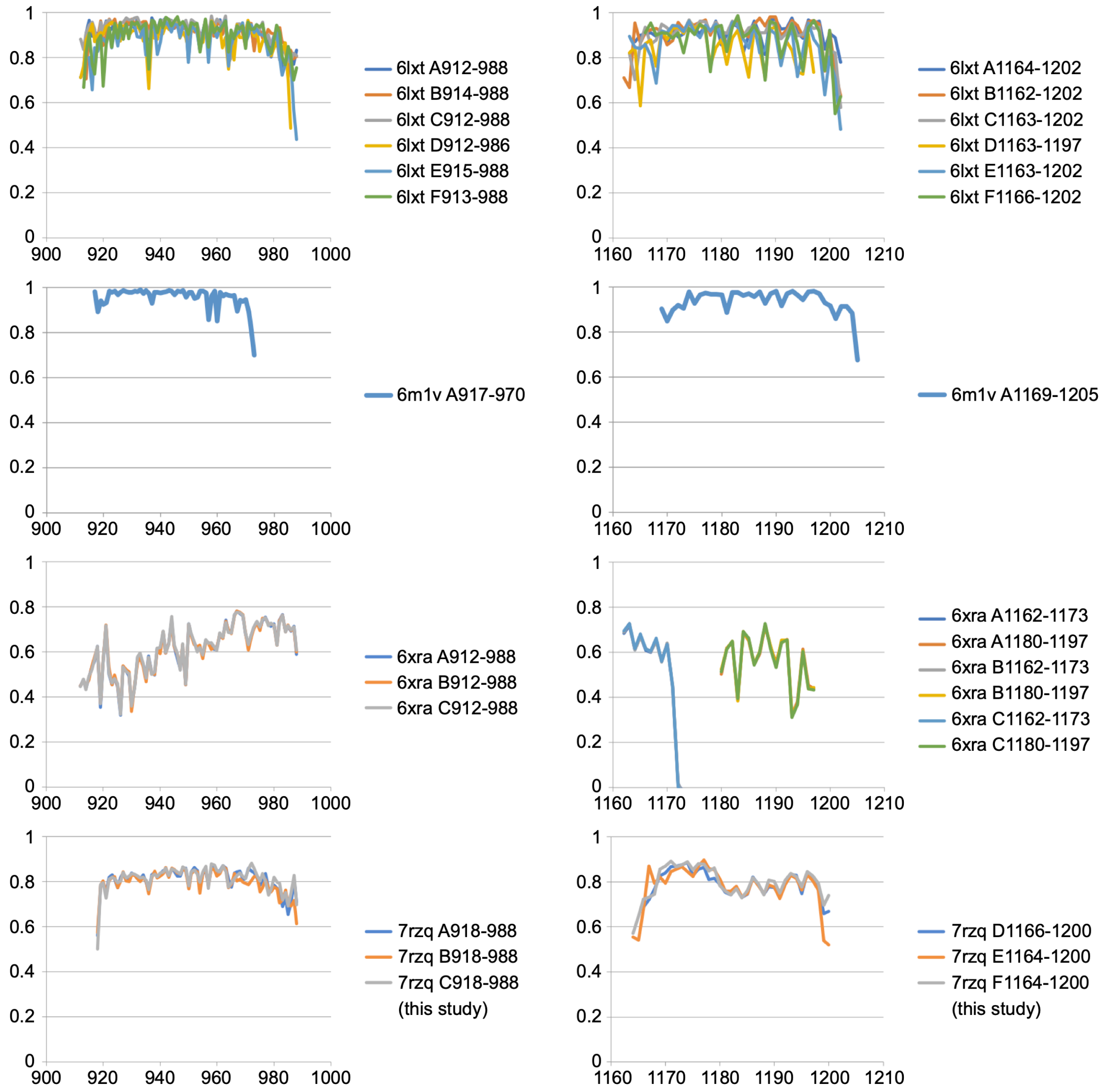


**Fig. S5.** Real-space cross-correlation between model and map of structures of HR1HR2 bundle of SARS-CoV-2. Per-residue real-space cross correlation scores (calculated with PHENIX (22)) are plotted on the Y axis and the residue number on the X axis. The PDB IDs, chain IDs, and residue numbers are indicated in the legends.

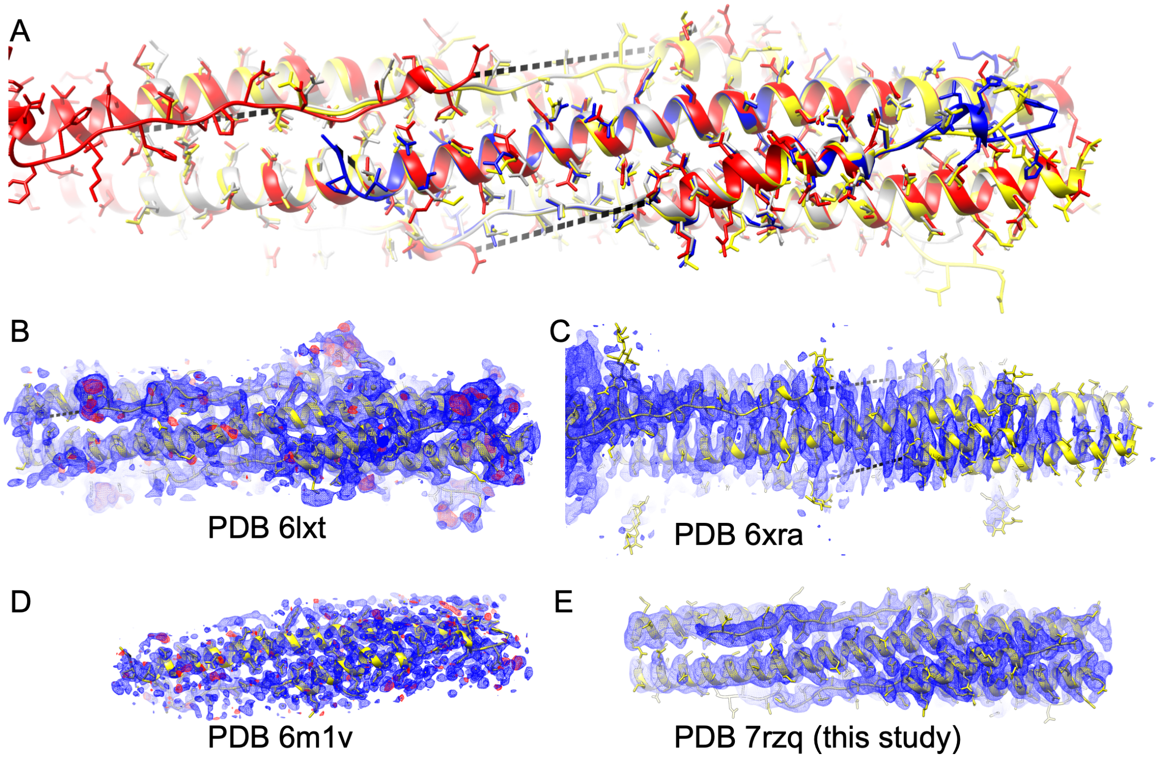


**Fig. S6.** Comparison of structures of SARS-CoV-2 post-fusion HR1HR2 bundle. (A) Superpositions of the wildtype HR1HR2 bundle structure (this study, PDB 7rzq, **Table S2**) (gray) with the crystal structure PDB 6lxt (yellow) (19), the crystal structure PDB 6m1v (blue) (23), and the EM structure PDB 6xra (red) (24). (B-E) Structures (yellow) and corresponding experimental maps ($2mF_o$-$DF_c$ crystal structure maps and EM maps are colored blue, $mF_o$-$DF_c$ crystal structure difference maps are colored red), displayed in the same orientation as in (A).

Table S1. Frequencies of mutations characterized in this study (top 6 mutations) or present in the common form of a SARS-CoV-2 variant of concern (VOC). The frequencies are obtained from the spike variant database (25, 26), as of Jan 16, 2022, that is based on a total number of ~5 million sequenced samples. For comparison, the total number of COVID-19 cases is ~330 million worldwide. A mutation is also noted as “compatible with Omicron” if it is found in some Omicron sequences.

| Mutation | Frequency | In a VOC? | Compatible with Omicron? |
|---|---|---|---|
| D936Y | 0.15% | No | Yes |
| L938F | 0.12% | No | Yes |
| S940F | 0.07% | No | Yes |
| A942S | 0.04% | No | Yes |
| V1176F | 2.23% | Gamma | Yes |
| Q954H/N969K/L981F | 1.38% | Omicron | Yes |
| D950N | 56.06% | Delta | Unknown |
| S982A | 19.77% | Alpha | Unknown |

**Table S2.** Cryo-EM data collection and processing

| | Wildtype | D936Y | L938F | S940F | A942S | V1176F | Omicron |
|---|---|---|---|---|---|---|---|
| Microscope | Titan Krios | Titan Krios | Titan Krios | Titan Krios | Titan Krios | Titan Krios | Titan Krios |
| Voltage (kV) | 300 | 300 | 300 | 300 | 300 | 300 | 300 |
| Camera | Gatan K3 | Gatan K3 | Gatan K3 | Gatan K3 | Gatan K3 | Gatan K3 | Gatan K3 |
| Pixel size (Å) | 0.653 | 0.653 | 0.653 | 0.653 | 0.653 | 0.653 | 0.65 |
| Exposure time (s) | 1 | 1 | 1 | 1 | 1 | 1 | 1.5 |
| Number of frames per exposure | 40 | 40 | 40 | 40 | 40 | 40 | 40 |
| Total Dose ($e^-/Å^2$) | 46 | 51 | 48 | 48 | 49 | 46 | 51.7 |
| Number of movies | 17,084 | 24,524 | 14,771 | 16,829 | 12,165 | 15,443 | 19,898 |
| Defocus range (μm) | -2 to -0.3 | -2 to -0.3 | -2 to -0.3 | -2 to -0.3 | -2 to -0.3 | -2 to -0.3 | -2 to -0.3 |
| Number of particles | 732,037 | 585,122 | 702,063 | 597,308 | 562,936 | 840,781 | 546,372 |
| Resolution of final global refinement (0.143 FSC, Å) | 1.82 | 1.8 | 1.82 | 1.83 | 1.69 | 1.82 | 1.77 |
| Resolution of final local refinement (0.143 FSC, Å) | 2.09 | 2.27 | 2.52 | 2.35 | 2.3 | 2.11 | 2.42 |
| EMDB | 24774 | 24775 | 24776 | 24777 | 24778 | 24779 | 25912 |

**Table S3.** Cryo-EM model building

| | Wildtype | D936Y | L938F | S940F | A942S | V1176F | Omicron |
|---|---|---|---|---|---|---|---|
| Bond RMSD (Å) | 0.006 | 0.007 | 0.005 | 0.005 | 0.006 | 0.005 | 0.007 |
| Angle RMSD (°) | 0.396 | 0.4 | 0.461 | 0.319 | 0.505 | 0.354 | 0.491 |
| Molprobity score | 1.24 | 1.11 | 1.09 | 1.33 | 1.54 | 1.29 | 1.75 |
| Clashscore, all atoms | 4.69 | 3.23 | 3.04 | 3.83 | 6.89 | 5.44 | 7.94 |
| Ramachandran favored (%) | 98.06 | 98.08 | 99.04 | 97.12 | 97.12 | 100 | 95.41 |
| Ramachandran allowed (%) | 1.94 | 1.92 | 0.96 | 2.88 | 2.88 | 0 | 4.59 |
| Ramachandran outliers (%) | 0 | 0 | 0 | 0 | 0 | 0 | 0 |
| Rotamer outliers (%) | 0 | 0 | 0 | 0 | 0 | 0 | 0 |
| Cβ outliers (%) | 0 | 0 | 0 | 0 | 0 | 0 | 0 |
| CaBLAM outliers (%) | 0 | 0 | 0 | 0 | 0 | 0 | 0 |
| PDB | 7rzq | 7rzr | 7rzs | 7rzt | 7rzu | 7rzv | 7tik |

**Table S4.** RMSD (Å) between the wildtype HR1HR2 structure presented in this work (PDB 7rzq) and other HR1HR2 structures

| PDB | Backbone atoms | Sidechain atoms | All atoms |
|---|---|---|---|
| 6lxt | 0.5 | 1.3 | 1.0 |
| 6m1v | 0.6 | 1.3 | 1.0 |
| 6xra | 1.0 | 2.0 | 1.5 |
| 7rzr (D936Y) | 0.2 | 0.8 | 0.6 |
| 7rzs (L938F) | 0.2 | 0.4 | 0.4 |
| 7rzt (S940F) | 0.2 | 0.4 | 0.4 |
| 7rzu (A942S) | 0.2 | 0.6 | 0.5 |
| 7rzv (V1176F) | 0.2 | 0.6 | 0.5 |
| 7tik (Omicron) | 0.8 | 1.2 | 1.0 |

**Supplementary Notes**

PDB codes of potential scaffolds considered in this study:
Trimer: 1fgj, 3b9w, 6eu6, 4d8m.
Hexamer: 4n2x, 4o0k, 4q0t.
Dodecamer: 2c41, 5hjf.
24-mer: 1fha.

The optimized construct for the scaffolded HR1:
MSHHHHHHGSQTLLRNFGNVYDNPVLLDRSVTAPVTEGFNVVLASFQALYLQYQKHHFVVEGS
EFYSLHEFFNESYNQVQDHIHEIGERLDGLGGVPVATFSKLAELTCFEQESEGVYSSRQMVEND
LAAEQAIIGVIRRQAAQAESLGDRGTRYLYEKILLKTEERAYHLSHFLAKDSLTLGFA**YENQKLIAN**
**QFNSAIGKIQDSLSSTASALGKLQDVVNQNAQALNTLVKQLSSNFGAISSVLNDILSRLDKVE***
(The HR1 fragment, corresponding to residues 917–988 of the spike protein, is in bold font.)

The optimized construct for the SUMO HR2:
MASLQDSEVNQEAKPEVKPEVKPETHINLKVSDGSSEIFFKIKKTTPLRRLMEAFAKRQGKEMDS
LRFLYDGIRIQADQAPEDLDMEDNDIIEAHREQIGGG**PDVDLGDISGINASVVNIQKEIDRLNEVA**
**KNLNESLIDLQ***
(The HR2 fragment, corresponding to residues 1162–1201 of the spike protein, is in bold font.)

**SI References**


1. V. M. Supekar *et al.*, Structure of a proteolytically resistant core from the severe acute respiratory syndrome coronavirus S2 fusion protein. *Proc Natl Acad Sci U S A* **101**, 17958-17963 (2004).
2. L. Lu *et al.*, Structure-based discovery of Middle East respiratory syndrome coronavirus fusion inhibitor. *Nat Commun* **5**, 3067 (2014).
3. L. Yan, B. Meng, J. Xiang, I. A. Wilson, B. Yang, Crystal structure of the post-fusion core of the Human coronavirus 229E spike protein at 1.86 A resolution. *Acta Crystallogr D Struct Biol* **74**, 841-851 (2018).
4. W. Weissenhorn, A. Carfi, K. H. Lee, J. J. Skehel, D. C. Wiley, Crystal structure of the Ebola virus membrane fusion subunit, GP2, from the envelope glycoprotein ectodomain. *Mol Cell* **2**, 605-616 (1998).
5. W. Weissenhorn, A. Dessen, S. C. Harrison, J. J. Skehel, D. C. Wiley, Atomic structure of the ectodomain from HIV-1 gp41. *Nature* **387**, 426-430 (1997).
6. X. Zhao, M. Singh, V. N. Malashkevich, P. S. Kim, Structural characterization of the human respiratory syncytial virus fusion protein core. *Proc Natl Acad Sci U S A* **97**, 14172-14177 (2000).
7. Z. N. Yang *et al.*, The crystal structure of the SIV gp41 ectodomain at 1.47 A resolution. *J Struct Biol* **126**, 131-144 (1999).
8. K. A. Baker, R. E. Dutch, R. A. Lamb, T. S. Jardetzky, Structural basis for paramyxovirus-mediated membrane fusion. *Mol Cell* **3**, 309-319 (1999).
9. Y. Xu *et al.*, Structural basis for coronavirus-mediated membrane fusion. Crystal structure of mouse hepatitis virus spike protein fusion core. *J Biol Chem* **279**, 30514-30522 (2004).
10. Z. Lou *et al.*, Crystal structures of Nipah and Hendra virus fusion core proteins. *FEBS J* **273**, 4538-4547 (2006).
11. Y. Liu *et al.*, Structural characterization of mumps virus fusion protein core. *Biochem Biophys Res Commun* **348**, 916-922 (2006).
12. S. Igonet *et al.*, X-ray structure of the arenavirus glycoprotein GP2 in its postfusion hairpin conformation. *Proc Natl Acad Sci U S A* **108**, 19967-19972 (2011).
13. J. F. Koellhoffer *et al.*, Crystal structure of the Marburg virus GP2 core domain in its postfusion conformation. *Biochemistry* **51**, 7665-7675 (2012).
14. J. F. Koellhoffer *et al.*, Structural characterization of the glycoprotein GP2 core domain from the CAS virus, a novel arenavirus-like species. *J Mol Biol* **426**, 1452-1468 (2014).
15. X. Zhang *et al.*, Crystal Structure of Refolding Fusion Core of Lassa Virus GP2 and Design of Lassa Virus Fusion Inhibitors. *Front Microbiol* **10**, 1829 (2019).
16. V. K. Outlaw *et al.*, Dual Inhibition of Human Parainfluenza Type 3 and Respiratory Syncytial Virus Infectivity with a Single Agent. *J Am Chem Soc* **141**, 12648-12656 (2019).
17. V. K. Outlaw *et al.*, Structure-Guided Improvement of a Dual HPIV3/RSV Fusion Inhibitor. *J Am Chem Soc* **142**, 2140-2144 (2020).
18. L. Belot *et al.*, Crystal structure of Mokola virus glycoprotein in its post-fusion conformation. *PLoS Pathog* **16**, e1008383 (2020).
19. S. Xia *et al.*, Inhibition of SARS-CoV-2 (previously 2019-nCoV) infection by a highly potent pan-coronavirus fusion inhibitor targeting its spike protein that harbors a high capacity to mediate membrane fusion. *Cell Res* **30**, 343-355 (2020).
20. E. F. Pettersen *et al.*, UCSF Chimera--a visualization system for exploratory research and analysis. *J Comput Chem* **25**, 1605-1612 (2004).
21. J. Pei, B. H. Kim, N. V. Grishin, PROMALS3D: a tool for multiple protein sequence and structure alignments. *Nucleic Acids Res* **36**, 2295-2300 (2008).
22. P. D. Adams *et al.*, PHENIX: a comprehensive Python-based system for macromolecular structure solution. *Acta Crystallogr D Biol Crystallogr* **66**, 213-221 (2010).
23. H. Sun *et al.*, Structural basis of HCoV-19 fusion core and an effective inhibition peptide against virus entry. *Emerg Microbes Infect* **9**, 1238-1241 (2020).
24. Y. Cai *et al.*, Distinct conformational states of SARS-CoV-2 spike protein. *Science* **369**, 1586-1592 (2020).

25. B. Korber *et al.*, Tracking Changes in SARS-CoV-2 Spike: Evidence that D614G Increases Infectivity of the COVID-19 Virus. *Cell* **182**, 812-827 e819 (2020).
26. B. Korber *et al.*, COVID-19 Viral Genome Analysis Pipeline. Retrieved Jan 16, 2022, from https://cov.lanl.gov/components/sequence/COV/int_sites_tbls.comp.
